# Stereotactic Arrhythmia Radioablation as a Novel Treatment Approach for Cardiac Arrhythmias: Facts and Limitations

**DOI:** 10.3390/biomedicines9101461

**Published:** 2021-10-13

**Authors:** Marina Chalkia, Vassilis Kouloulias, Dimitris Tousoulis, Spyridon Deftereos, Dimitris Tsiachris, Dimitrios Vrachatis, Kalliopi Platoni

**Affiliations:** 1 Radiotherapy Unit, Second Department of Radiology, School of Medicine, Rimini 1, National and Kapodistrian University of Athens, 124 62 Athens, Greece; vkouloul@ece.ntua.gr (V.K.); polaplatoni@gmail.com (K.P.); 2 First Department of Cardiology, ‘Hippokration’ General Hospital, Vasilissis Sofias 114, 115 27 Athens, Greece; tousouli@med.uoa.gr; 3 Second Department of Cardiology, “Attikon” University Hospital, School of Medicine, Rimini 1, National and Kapodistrian University of Athens, 124 62 Athens, Greece; spdeftereos@med.uoa.gr (S.D.); dvrachatis@gmail.com (D.V.); 4 Athens Heart Center, Distomou 5-7, 151 25 Athens, Greece; dtsiachris@yahoo.com

**Keywords:** ventricular tachycardia, atrial fibrillation, cardiac arrhythmias, radioablation, stereotactic radiotherapy, stereotactic body radiation therapy, quality assurance, dosimetry, respiratory gating

## Abstract

Stereotactic ablative radiotherapy (SABR) is highly focused radiation therapy that targets well-demarcated, limited-volume malignant or benign tumors with high accuracy and precision using image guidance. Stereotactic arrhythmia radioablation (STAR) applies SABR to treat cardiac arrhythmias, including ventricular tachycardia (VT) and atrial fibrillation (AF), and has recently been a focus in research. Clinical studies have demonstrated electrophysiologic conduction blockade and histologic fibrosis after STAR, which provides a proof of principle for its potential for treating arrhythmias. This review will present the basic STAR principles, available clinical study outcomes, and how the technique has evolved since the first pre-clinical study. In addition to the clinical workflow, focus will be given on the process for stereotactic radiotherapy Quality Assurance (QA) tests, as well as the need for establishing a standardized QA protocol. Future implications and potential courses of research will also be discussed.

## 1. Introduction

Cardiovascular diseases represent the leading cause of death globally. It is estimated that approximately 17.9 million people die from cardiovascular diseases each year, accounting for 31% of the global death toll [1]. Ventricular arrhythmias (VAs) may occur in the context of structural or ischemic heart disease causing significant morbidity and mortality. The most common VA is the monomorphic ventricular tachycardia (VT) [2,3]. VT is a life-threatening arrhythmia often caused by electric reentry within and around patches of heterogeneous myocardial fibrosis or scar [4]. Current therapeutic quiver involves (a) conservative drug treatment and/or transcatheter ablation techniques as a means of VA primary prevention and (b) implantation of devices (defibrillators) for secondary prevention. Ablation procedures involve introduction of special catheters into heart chambers in order to apply radiofrequency or cryoablation treatments to the myocardium.

However, the most common arrhythmia in the Western world is atrial fibrillation (AF) which does not originate from the ventricles but from the left atrium. AF is, indeed, a growing global health concern with a 19% increase over the last 20 years and 5 million new cases diagnosed each year worldwide [5]. Hence, it has been characterized as a silent pandemic. Currently, it is thought that an abnormal electrical communication of left atrial myocardium and pulmonary veins is, at least partially, responsible for AF [3]. Accordingly, currently, interventional treatment for AF in view of maintenance of sinus rhythm is pulmonary vein isolation. Peripheral vascular vein access is required in order to advance special catheters (radiofrequency; cryoballoon; laser balloon) into the left atrium and apply ablation treatments.

As already stated, available therapeutic options for VA include implantation of implantable cardiac defibrillators (ICDs), antiarrhythmic drugs and/or interventional therapies, namely catheter ablation. Each therapeutic means may entail significant side effects. ICDs are highly effective in reducing the rate of sudden cardiac death due to ventricular tachyarrhythmia among high-risk cardiac patients. However, inappropriate ICD activation, typically caused by supraventricular tachyarrhythmias, are still a significant issue, despite sophisticated detection algorithms designed to differentiate supraventricular from ventricular tachyarrhythmias; such activations adversely affect outcomes [6]. Further, recurrent arrhythmias and ICD shocks may cause impairment in the quality of life, while multiple shocks are associated with post-traumatic stress disorder [7]. Antiarrhythmic drug therapy targeting these substrates has been available for many years, but is often ineffective, with multiple untoward effects, and may even enhance arrhythmogenesis if not used prudently [3]. In particular, amiodarone therapy has been shown to reduce recurrent arrhythmias in the first year of treatment by 71% as well as arrhythmia-related death, but it has been associated with a substantial risk of side effects during long-term therapy [6]. The VANISH trial evaluated escalated antiarrhythmic drug therapy vs. catheter ablation and showed that the latter was more effective in reducing the rate of the combined outcome of death at any time or ventricular tachycardia storm or ICD shocks at 30 days [6]. Catheter ablation delivers unipolar radiofrequency (RF) energy to eliminate the re-entry circuit responsible for VT. Notably, there are a few instances in which catheter ablation fails, especially in VTs of a deep intramural origin or cases in which epicardial access is not feasible. Of note, Tokuda et al., in a retrospective study, reported ablation failure in approximately 10% of patients undergoing VT ablation [8]. Indeed, the decision for redo procedures is usually based upon local or operator-specific policies without sound evidence to ground on.

Similarly, in the case of AF, specialized catheters are introduced into the atria via the venous vasculature to create scar and disrupt the postulated underlying arrhythmogenic substrate (i.e., electrically isolate pulmonary veins from left atrium) [9]. Success rates and complication risks vary between procedure types, highly dependent upon the underlying substrate targeted [3]. However, some patients may need multiple catheter ablation procedures. Hence, there is a clear clinical need to delineate the optimal approach in patients in need of a redo ablation procedure [10]. Indeed, pulmonary vein reconnection and return of electrical activity in the ablation target has been reported after initially successful AF ablation [3].

To overcome limitations of transcatheter ablation approach, several alternative approaches have been explored, including alcohol ablation, coil embolization, simultaneous unipolar or bipolar RF ablation, surgical ablation, or, lately, noninvasive ablation with stereotactic radiosurgery [11]. Stereotactic arrhythmia radioablation (STAR) boasts elimination of access site complications and ability to target virtually any cardiac site of interest. However, several hurdles need to be overcome, mainly ensuring adequately compensation for target movement and achieving delivery of appropriate energy to achieve suitable lesion formation while protecting adjacent tissues, before this method can be widely applied. These aspects of STAR will be reviewed in the present article.

## 2. Materials and Methods

### 2.1. Literature Search

**Figure 1:**
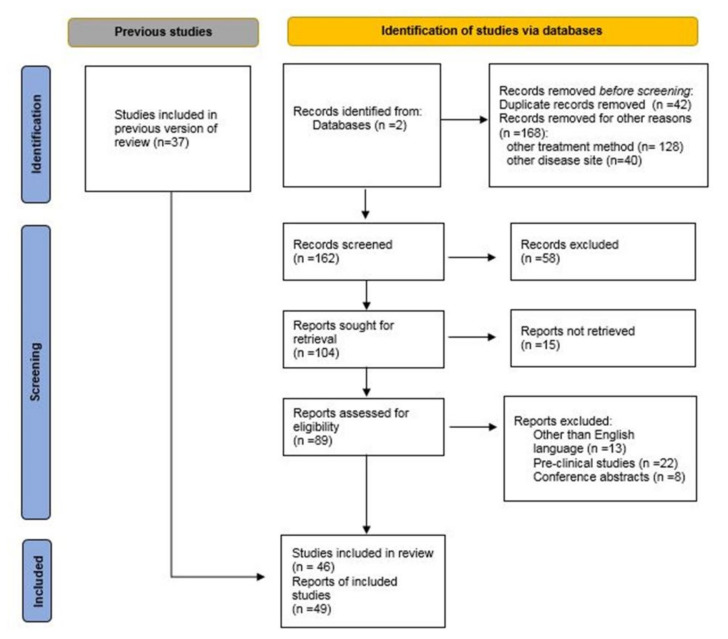
PRISMA flow diagram for the review which includes searches of databases.

A comprehensive literature search in PubMed and ScienceDirect for the following query “ventricular tachycardia; atrial Fibrillation; cardiac arrhythmias; radioablation; stereotactic radiotherapy; stereotactic body radiation therapy; quality assurance; dosimetry; respiratory gating”, revealed a total of 39 original clinical articles and 7 reviews, which provided data for the implementation of stereotactic ablative radiotherapy (SABR) for cardiac arrhythmias, either VTs or AF. The articles chosen for evaluation provided sufficient information for any aspect of the procedural workflow (pre-treatment imaging, motion management, treatment planning, quality assurance, treatment delivery). For the quality assurance processes and guidelines for stereotactic radiation therapy, publications from 2006 to 2021 were included. For the clinical processes for cardiac radioablation, no date restriction was applied. The literature search was conducted on 30 April 2021. Articles not in the English language and publications regarding pre-clinical data were excluded. Figure 1 shows the flow diagram of the review literature search, according to PRISMA guidelines [12].

### 2.2. Stereotactic Arrhythmia Radioablation: Basic Principles

SABR was first used to treat brain tumors with a goal of delivering high therapeutic doses to the intended target tissue while minimizing doses to adjacent normal anatomic structures [13]. With the ongoing advances in radiotherapy, such as imaging, tracking and intensity modulation, the use of SABR has been expanded to other organs. STAR has been recently investigated for ablation of the critical part of the arrhythmogenic substrate in cases of failed or contraindicated catheter ablation. STAR has the potential to deliver ablative energy noninvasively, minimizing any procedural risks, especially the access vessel associated ones [14]. While fractionated irradiation leads to tumor cell, primarily through formation of DNA double-strand breaks, the mechanism of injury for high doses of radiotherapy is less well known. A possible mechanism is a combination of vascular injury, leading to tissue hypoxia or necrosis, and apoptotic cell death, resulting in fibrosis and scar formation [3]. The field is vaguer as far as cellular injury mechanisms that may be specific to the nonmalignant arrhythmogenic cardiac tissues are considered. Compared to RF ablation, which features its antiarrhythmic potential acutely, SABR requires days to months to manifest the scar injury in the tissue of interest. Indeed, early preclinical and clinical experience with STAR suggests safety and efficacy of STAR after its application [3].

#### 2.2.1. Patient Characteristics

The first STAR patient with VT was treated at Stanford University on a robotic radiosurgery system (CyberKnife^®^, Accuray, Sunnyvale, CA, USA) in 2012 [2]. To date, there have been reported 68 patients treated with STAR (published data); 52 VT patients within clinical trials [4,15,16,17,18,19,20]; 10 VT patients within clinical single-case series [21,22,23,24,25,26,27,28,29,30], and 6 AF patients [9,31,32]. Among treated VT patients, 34 have been treated with the Varian Linac system, and the rest of them have been treated with the Accuray CyberKnife system.

STAR holds the promise of being an efficient and safe therapeutic option for many patients with cardiac arrhythmias, however, currently, this modality has been only utilized as second line option, in patients in whom the conventional approach has failed. Patients were evaluated on a case-by-case basis for inclusion in the STAR protocol. Table 1 summarizes characteristics of patients with VT as well as inclusion/exclusion criteria followed by each study. The vast majority of patients were males (>70%), with a mean age of 65 years; range 29–75 years. The common inclusion criterion adopted by all studies was catheter ablation failure. Most patients remained unresponsive to catheter ablation and suffered from incessant VTs, or the target myocardial segment was inaccessible, thus redoing catheter ablation was not deemed appropriate. Patients supported under mechanical assist devices, specifically left-ventricular assist device (LVAD), were excluded from STAR protocols [4,15,16], because of the potential effect of radiation on the function of the LVAD system [33]. All included patients are reported to have provided informed consent; study protocols were approved by local institutional ethic boards.

#### 2.2.2. Assessment of Treatment Efficacy and Safety

**Figure 2:**
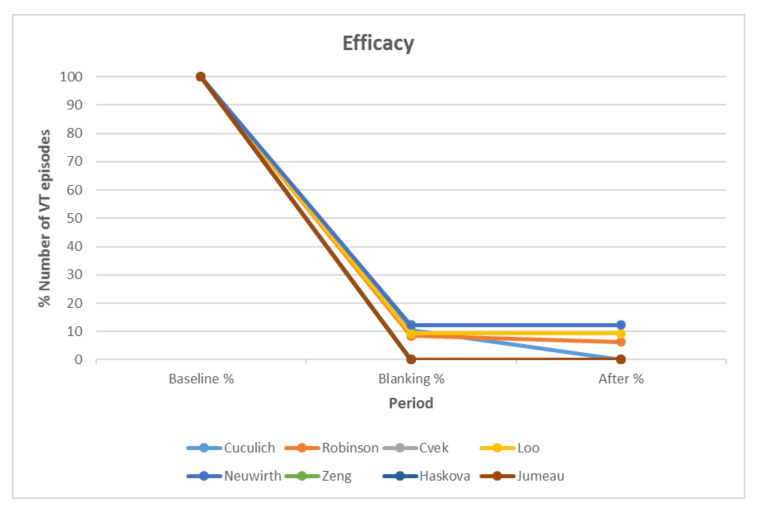
Percentage (%) of Ventricular Tachycardia (VT) episodes, as recorded by Implantable Cardioverter-Defibrillators (ICDs), according to 8 Stereotactic Arrhythmia Radioablation (STAR) studies. Baseline refers to certain observation period before treatment. The VT episodes are expressed as a percentage of the absolute number of VT episodes at baseline (baseline VTs = 100%), as detected by ICDs. The studies of Cuculich and Robinson provide data for the VT episodes during the blanking period. For the other studies, equal number of VT episodes during blanking and after therapy period is assumed. Cvek, Zeng, Haskova, and Jumeau record the same % number of VT episodes (from 100% to 0%).

Regarding VTs, the primary efficacy end point (defined as any reduction in episodes as monitored by ICDs) has been demonstrated in almost all treated patients [3,17,25,26]. Still, two studies reported three patients who showed practically no treatment effect [16,17]. One of these patients underwent a second ineffective SABR treatment 90 days later and ultimately underwent heart transplantation [19]. Regarding long-term efficacy, a pilot prospective study of 5 patients concluded that STAR showed good initial results, but did not result in effective arrhythmia control in the long term [19]. A few other studies report VT recurrence >6 months after therapy in 7 of 15 evaluable patients [15,16,22]. For AF treatment, STAR demonstrated electrophysiological and histological effect to the left atrium [9,31,32]. Patients’ follow-up, usually up to 12 months after STAR, was conducted on the basis of ECG monitoring and ICD interrogation. The electrophysiologist, along with the radiation oncologist evaluated echocardiographic data and the number of VT episodes, remotely and at each study visit, and chest Computed Tomography (CT) in order to assess the safety and efficacy of treatment. Figure 2 shows the percent number of VT episodes, as recorded by ICDs, before treatment, during the blanking period, and after treatment for 8 studies.

Pre-STAR monitoring period ranged from 24 h to 6 months. The blanking period was 1.5–3.0 months long and the follow-up period lasted from 2 to 54 months. Almost all studies report a significant reduction or even disappearance of VT episodes after STAR. Patients were administered standard antiarrhythmic treatment before STAR, mainly amiodarone, mexiletine, and sotalol. Some studies report reduction of antiarrhythmic medication after STAR, while, in a number of patients, complete medication withdrawal was feasible several weeks after treatment [15,18].

Beyond antiarrhythmic effect, STAR treatment has also been reported to improve left ventricular ejection fraction. In particular, Robinson reports such an improvement in 72% of treated patients [4]. Further, overall quality of life was also assessed, utilizing the RAND Short Form-36 questionnaire. Improvements in self-perceived change in health status and social functioning categories, or general stability after treatment were observed [4,22].

Regarding safety, the primary safety end point was treatment-related serious adverse events (SAE). Common Terminology Criteria for Adverse Events (version 4.0) that were treatment-related (possibly, probably, or definitely) was adopted by some trials [4,16,34]. Acute and late radiation-induced events were organized by system; e.g., cardiac, gastrointestinal, immune system or respiratory disorders, and then further divided into specific adverse events [4]. Table 2 summarizes the safety end points of each study and the monitoring methods being selected. Most studies report no complications during the treatment or the follow-up period. Among all treated patients, two developed a treatment-related SAE, namely delayed pericarditis/effusion and pneumonitis, which were generally responsive to medical therapy [4], the other two experienced mild clinical and radiographic signs of pneumonitis [17], while the only acute radiation-related toxicity observed was nausea of 5 patients [16,27].

### 2.3. STAR Workflow and Treatment Delivery

**Figure 3:**
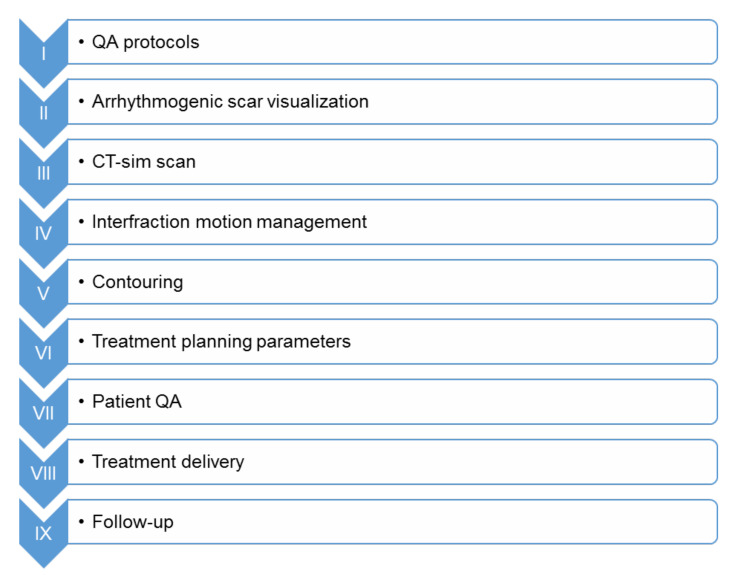
Stereotactic Arrhythmia Radioablation (STAR) procedural workflow. Prior treatment and the correct identification of the anatomic scar is important. QA: Quality Assurance.

As expected for such a novel approach, each study has reported its own distinct procedural steps, based on the department’s available technology. However, the main roadmap to perform STAR remains constant. Figure 3 depicts the procedural workflow for optimal STAR application. Each link of this chain is important for the successful treatment application and will be analyzed in detail.

#### 2.3.1. Quality Assurance Processes

Unlike conventional radiotherapy, where the radiation is fractionated into small dosages delivered over weeks, Stereotactic Body Radiation Therapy (SBRT) uses numerous non-coplanar beamlets that converge on a single target volume to deliver high-dose radiation. The high target dose is being delivered with sub-millimeter accuracy and from different beam angles, minimizing injury to adjacent organs [14]. Given the high dose in small target volumes and rapid dose fall-off, it is important to reduce uncertainties in the target delineation and localization. The success of SBRT relies largely on image-guidance radiotherapy (IGRT) methods and advanced dosimetric quality assurance (QA) procedures.

Thus, prior STAR treatment application, the physicists’ group should develop and implement an appropriate QA protocol. An end-to-end test should be performed to identify whether the flow of STAR treatment is running as designed from start to finish. All aspects of the treatment process are tested, including imaging methods, immobilization devices, simulation process, respiratory and cardiac-related motion management, treatment planning, and treatment delivery. Each step in the end-to-end testing is being performed by the clinical team members who will perform the step, when the program is clinically implemented. Departments performing STAR treatments should be equipped with the appropriate resources, referring to both staff and equipment. Several medical physicist groups have published guidelines for an appropriate QA process in SBRT [35,36,37]. A comprehensive QA program includes the following critical components:

##### Treatment Delivery Machine Evaluation

The equipment performance is checked in order to be within tolerance levels. The minimum SBRT relevant machine QA tests include:

- Small-field measurements, performed with appropriate stereotactic detectors (spatial resolution of approximately 1 mm, or better). International Atomic Energy Agency has published a code of practice for small field dosimetry, which is a useful guideline [38];
- Laser localization test;
- Radiation isocentricity test (STAR pattern), covering complete range of gantry, couch, collimator positions used clinically;
- IGRT positioning/repositioning;
- Accelerator output constancy;
- Coincidence of radiation and mechanical isocenter. The Winston-Lutz test is commonly used to establish coincidence of planning and delivery isocenters [39];
- End-to-end assessment of geometric accuracy of the entire imaging, planning, and delivery process using dosimetry phantoms and IGRT systems. The basic phantom is a simple block of plastic with small, embedded ball bearings positioned as fiducial markers. A more elaborate procedure could be with an anthropomorphic phantom with fiducial markers placed at selected locations to simulate registration landmarks and target locations [40];
- End-to-end dosimetric evaluation using dosimetry phantoms and IGRT systems.

For robotic radiosurgery systems, TG-135 provides specific QA guidance [37]. Except for the above mentioned, some extra tests include:

- IrisTM field-size spot check, for CyberKnife systems;
- Path verification;
- Beam laser and radiation beam alignment for cone, IrisTM, and multi-leaf collimator (MLC).

Detailed documentation for the QA processes is beyond the scope of this article.

##### Immobilization Devices and Simulation Procedure

Immobilization devices should be evaluated for their effectiveness in targeting precision. Position shifts should be minimized, by keeping patients at the same position, but also making them feel comfortable during the whole treatment time. Immobilization equipment should also be evaluated for its beam attenuation and surface dose characteristics, prior to clinical implementation.

For the CT-simulation, reproducible immobilization techniques should be developed. The imaging CT to be used for treatment planning should cover the target and all relevant organs at risk. A typical scan length should extend at least 10 cm beyond the treatment field borders to adequately model the beam paths and resultant scatter dose [36]. CT slice thickness of 1–3 mm should be used and the field of view should be optimized for maximum in-plane spatial resolution, while including all necessary immobilization devices. For STAR technique, CT slice thicknesses of 1, 1.25, 1.5, and 3 mm have been used [4,15,17,18,19,41]. Respiratory and cardiac motion management should also be considered.

##### Motion Management

Organ motion due to free breathing creates additional concerns related to target precision. Due to STAR treatment site, which is affected by both respiratory and cardiac motion, the entire treatment chain (CT simulation, treatment planning, and treatment delivery) should be assessed with end-to-end testing using a dynamic phantom setup with clinically relevant motion parameters (amplitude, cycle time). The tests should include assessment of spatial targeting accuracy and measurement of delivered target dose [36]. Interfraction motion management techniques involve respiratory gating and target tracking. Gating techniques fully encompass the movement of the target and the dose is delivered only when the target is within a defined region. This technique requires four-dimensional CT imaging to define the target motion. Respiratory tracking techniques, track the moving target throughout the cycle. CyberKnife uses the Accuray Synchrony Respiratory Tracking System, where a robotically-mounted linear accelerator can redirect the radiation beam to follow the moving target. Varian uses a respiratory gating system, the Real-time Position Management (RPM) or the most recent Respiratory Gating for Scanners (RGSC), which has four different scanning modes available: “4D Scan”, “Phase Gating”, “Amplitude Gating” and “Breath-hold Gating”. The RGSC system is superior to the RPM system, especially for irregular breathing patterns, probably due to improved camera resolution and better predictive functionality from the software design [42]. Most studies use the “4D Scan” during the CT simulation process for STAR [4,15,17,18,19,20,21,22,23,24,25,26,27]. Oftentimes, a fiducial marker may be localized near the cardiac target and used to image movement with X-ray or CT imaging. Potentially, any radiologically discrete structure that moves in tandem with the target can be used. However, several groups have performed STAR effectively without temporary fiducial placement [15]. In this case, intracardiac and extracardiac native or implanted structures can also be used as a fiducial marker. Specifically, coronary stents, mechanical valves, ICD pacing leads, the diaphragm, or even rib bony borders can be used.

The important QA step needed for either respiratory gating or target tracking is the use of phantoms that move with a reciprocating platform to simulate respiratory motion [40]. These phantoms should allow appropriate detectors, such as ionization chambers, diodes or films, to be attached during motion, so that absolute dosimetric measurements can be made and can demonstrate that a tracking or gating system performs within acceptable limits. Varian has used the QUASAR Respiratory Motion Phantom, a dynamic 4D phantom, with a sinusoidal breathing cycle of a 5 s period and a mathematical fitting method for data acquisition and analysis of the gating system [42]. The AAPM Task Group 76 report describes a QA program for assessing and managing respiratory motion [43].

##### Treatment Planning System

The treatment planning system should accurately calculate the predicted dose for the scope of SBRT plans. Target dose coverage, dose gradient, dose conformity metrics, and organs-at-risk dose objectives should be clearly reported and signed by the radiation oncologist to confirm that the chosen treatment technique is clinically acceptable. Some clinical studies use extra-cardiac organs-at-risk (OAR) dose constraints based on hypo-fractionated centrally located lung cancer treatments and TG101 technical specifications for SBRT treatments [5,17,35,44]. Most extra-cardiac OAR dose constraints are easily achieved in clinical studies for VT radioablation [20,27,43], in contrast to AF treatments where it is most challenging to meet esophageal dose constraint [5,34]. Blanck et al. generated theoretical AF radiosurgery treatment plans for twenty-four patients. For AF radioablation with 25 Gy under ideal tracking conditions and a 3 mm safety margin, they concluded that the treatment may only be feasible in less than 40% of the patients. This is mainly due to the unfavorable esophagus and bronchial tree location relative to the left atrial antrum (target area) [34]. For intra-cardiac structures, there is no clear consensus recommendations for STAR dose constraints. Knutson et al. published a dosimetric analysis of the targets, OARs, and cardiac substructures for 16 patients [20]. They reported median doses, interquartile range (IQR), and equivalent dose in 2 Gy fractions (EQD2) for a large range of intra-cardiac substructures.

In another study, Wang et al. compared dosimetrically three possible treatment delivery platforms for STAR [2]; CyberKnife^®^ G4 system with Iris collimator (Multiplan, V. 4.6) (Plan #1), CyberKnife^®^ M6 system with InCise 2TM multi-leaf collimator (Multiplan V. 5.3) (Plan #2), and Varian TrueBeamTM STx with HD 120TM MLC and 10 MV flattening filter free (FFF) beam (Eclipse planning system, V.11) (Plan #3 coplanar and #4 noncoplanar volumetric modulated arc therapy (VMAT) plans). The four plans were compared by prescription isodose line, plan conformity index, dose gradient, dose to the nearby critical structures, planned monitor units (MU), and estimated treatment time. Clinically acceptable plans were created with all three platforms. CyberKnife^®^ MLC plan had the best dose gradient, while the TrueBeamTM STx co-planar plan the worst. The dose to nearby critical structures (lung, stomach, bowel, and esophagus) were all well within tolerance. VMAT plans were most efficient in MUs and total-treatment-time/beam-delivery-time. Specifically, the MUs for plans #1–4 were 27,671, 16,522, 6275, and 6004 for an estimated total-treatment-time/beam-delivery-time of 99/69, 65/35, 37/7, and 56/6 min, respectively. A pretreatment setup time of 30 min was assumed and for VMAT gated delivery, a 40% duty cycle. An extra 10 min was given per extra arc, due to the fact that the patient localization will need to be verified again and the gating window reset. Total treatment time is much longer than the beam-on time due to target tracking method (IGRT—Image Guided Radiation Therapy). CyberKnife plans are more time consuming due to continuous fiducial imaging, while VMAT plans with Varian TrueBeam/Edge systems use X-rays taken at regular intervals throughout treatment to correct the patient position [14].

Furthermore, the medical physicist should ensure that the radiation oncologists are aware of the delivery system’s tolerances relative to the planning target volume and organs-at-risk avoidance margins [40]. Image fusion requirements for target delineation should be defined and target margins should be clearly described.

#### 2.3.2. Imaging the Arrhythmia Substrate

STAR treatment planning and application at a radiotherapy department requires the smooth collaboration of a multidisciplinary team, including radiation oncologists, cardiologists, physicists, and therapists. Prior treatment of the correct identification of the arrhythmogenic scar is important in order to delineate an accurate 3-dimensional (3D) target volume. This can be achieved through anatomic imaging modalities in combination with electrophysiological information. Patients undergo cardiac imaging to identify regions of anatomical scarring with CT, single-photon emission computerized tomography (SPECT) or contrast-enhanced cardiac Magnetic Resonance Imaging (MRI) [4,15]; 12-lead electrocardiography (ECG) is a simple tool that can localize the origin of VT/premature ventricular contractions (PVCs), or even some atrial tachycardia substrates with good accuracy [45]. However, it has very limited spatial resolution for determining regional cardiac electrical activity and limited ability to locate regions of arrhythmic activity in the heart [46]. Due to this limitation, most departments perform 3D electroanatomic mapping (EAM) produced by previously undergone catheter ablation procedure. Small electrodes are guided into the heart and record signals directly from the myocardium, providing valuable information for the areas of arrhythmic activity being studied. The system acquires these data and creates a 3D mesh of points with the values of interest attached to each point. There are currently several commercially available catheter-based systems that can be used to obtain this data, including CARTO3 (Biosense Webster), Rhythmia (Boston Scientific), Topera (Abbott), and EnSite (Abbott) [16,21,25,47].

Electrocardiographic imaging (ECGI) is an alternative noninvasive CT-based method for electroanatomic mapping of cardiac electrical activation. ECGI combines body-surface potentials and patient-specific heart-torso geometry to noninvasively construct the local electrical signals of the heart over the entire surface of both the ventricles [46]. Gated CT scans are being performed in order to obtain the heart-torso geometry. ECGI has the ability to map the VT circuit from a single beat. ICDs are used in order to induce VT and to terminate VT with a brief overdrive-pacing maneuver, during the ECGI procedure. After programmed stimulation, the electrical and anatomical data are processed mathematically to obtain noninvasive ECGI epicardial images that include potential maps, electrograms, and isochronal activation sequences. Clinical results using ECGI in combination with anatomical imaging have been very promising to identify the arrhythmia exit site [4,15,20,46].

Each department applies its own technique and software program for the ECGI procedure. Cuculich et al. used a vest of 256 electrodes with small radiopaque markers attached at the location of the electrodes (BioSemi, Netherlands) to assist with visualization on cardiac imaging [15]. Wearing the vest, patients underwent a gated chest CT scan to provide heart-torso geometry and torso-electrode positions in the same reference frame.

#### 2.3.3. CT-Simulation

Before treatment, patients undergo a planning CT-simulation scan, which is an imaging procedure intended to simulate the patient’s position at the time of treatment delivery. The simulation process includes the creation of a custom immobilization device for the patient, covering the entire body from thorax to legs. The device allows the patient to be positioned on the treatment table in a manner that is identical to their position at the time of imaging. The patient is placed supine on the treatment table and made comfortable to minimize patient movement for the duration of treatment delivery. A vacuum-assisted cushion, shaped to the patient’s body, is frequently used [4,15]. Abdominal compression can also be used to decrease the movement of target locations [4]. One study used no vacuum bag or abdominal compression, due to the poor clinical condition of the patient [27].

Once the custom immobilization device is made, a series of CT scans are acquired with the proper beam parameters. A free-breathing and a respiration-correlated CT (4D-CT), which provides information about the sum total of cardiac and pulmonary motion, are acquired in studies without fiducial guidance [3]. The free-breathing planning CT is obtained to serve as the anatomic reference upon which a 3D target is contoured. The 4D-CT is comprised of a series of reconstructed CT scans corresponding to different breathing phases and provides information about target position throughout the full respiratory cycle. A composite of these scans is fused with the free-breathing planning CT to create an adjusted planning image set [14]. The irradiation may then be delivered via respiratory gating or a breath-hold technique [3]. For treatments utilizing fiducial tracking, planning CT was performed in expiratory breath-hold with intravenous contrast enhancement and target position was instead evaluated during the treatment delivery process [16,19].

#### 2.3.4. Interfraction Motion Management

Heart is a moving organ affected by both the respiratory motion and the cardiac contraction, which adds an additional level of complexity in treatment planning. Cardiac motion is much less than the pulmonary one, especially in STAR patients, who have reduced contractility, therefore, some studies consider that respiratory motion accounts for the vast majority of target motion [3]. The degree of cardiac motion is affected by the location of the target and the disease state of the patient. Basal locations in the heart and large regions of existing scar reduce target motion [41].

Respiratory interfraction motion management may be accomplished with:

- Inhibition, which aims to limit respiratory motion using abdominal compression or breath-hold technique;
- Gating, which follows the full respiratory cycle to deliver radiation only when the target is within a defined region of the cycle. Varian Medical Systems implement the RPM and the RGSC respiratory gating systems. TG-147 provides detailed information for the test of integration of the respiratory gating device with peripheral equipment, spatial reproducibility and drift, static and dynamic localization accuracy [48];
- Tracking methods, which redirect the radiation beam to follow the moving target. Fiducial markers serve as surrogates for the target position. Fiducial markers may be an existing device component, an implanted gold seed or intracardiac and extracardiac structures. CyberKnife synchronizes the moving robotic arm with the target in real-time using an internal respiratory tracking system (Synchrony Respiratory Tracking System). The respiratory tracking system uses continual imaging of fiducials to align the radiation beam with the motion of the target.

To additionally assess cardiac motion, cardiac-gated CT scans in both systole and diastole [16,19,40,49], or fluoroscopy imaging of ICD leads during transient breath-holds have been used [22,23].

#### 2.3.5. Contouring

All patients underwent pre-procedural imaging to delineate target volume. Those with prior EAM had their maps incorporated into the treatment planning system (TPS). The sum total of electrophysiology information (ECG, EAM, ECGI) and the anatomical information (SPECT, MRI, CT, and/or echocardiogram) was combined to generate a probability mask for the arrhythmogenic substrate, meaning the target for radioablation (gross target volume—GTV). For the delineation of Internal Target Volume (ITV), the majority of c-arm linear accelerator treatments used a combined cardio-respiratory ITV derived from free-breathing or motion-compressed respiratory-gated 4D-CT to account for the maximum range of motion [4,15,17,20,26,30,34]. Heart movement was encompassed in the ITV delineation by several groups [2,22,23]. Specifically, Wang et al. included a 5 mm margin for the ITV to compensate for heart movement [2]. Nearby normal organs including the lung, bowel, esophagus, and stomach were also delineated with the appropriate margins. Gianni et al. used a pre-defined 3 mm margin expansion in all patients as an ‘average’ cardiac motion ITV [19], while Jumeau et al. used no ITV after evaluating the low degree of cardiac motion with transthoracic echocardiography [25].

The final planning target volume (PTV) was developed by expanding the target to account for motion, setup uncertainty, and delivery uncertainty. The volumes of the final PTVs depend on the imaging modalities, the image fusion procedure, motion management, treatment delivery unit, and the target margins adopted by each group. Early CyberKnife treatments did not utilize additional PTV margins [16,21,25], while later CyberKnife treatments utilized 3 mm isotropic PTV margins [33,49,50]. C-arm linear accelerator treatments utilized mostly 5 mm isotropic PTV margin (range 1–8 mm) [4,15,17,20,26,27]. Robinson et al. published a study of 19 patients in Varian system whose GTV delineated, ranged from 6.4 to 88.6 cc, while the PTV ranged from 60.9 to 298.8 cc [4]. Cuculich and Neuwirth published data with smaller PTVs ranging from 14.2 to 81 cc [15,16].

#### 2.3.6. Treatment Planning Parameters

Nearly all clinical STAR treatments delivered 25 Gy as a single fraction. The prescribed radiation dose of 25 Gy was delivered at the 75% or 80% isodose line, for the CyberKnife system [16,22,24]. For AF treatments, there are studies which demonstrate that 25 Gy can only be considered safe if (a) 25 Gy is indeed the minimum treatment effective dose, (b) the target volume margins can be kept at 3 mm or lower, and (c) the esophagus is centrally located at the left atrium posterior wall and does not move during treatment [5,34]. On the other hand, for VT treatments, 25 Gy seem to be much easier to apply due to (a) larger distance between the target and radiosensitive critical structures, (b) limited cardiac and differential motion (smaller uncertainty margins possible), (c) fiducial marker already near or in the target area [49]. Zeng et al. delivered 24 Gy in three fractions [23]. Chin et al. performed STAR with doses of 15–25 Gy, and reported also one patient who received a second round of SBRT due to recurrent VT originating from a distinct and distant left ventricular segment [18]. In preclinical studies, higher dosing was found to be safe [11,51]. Conclusively, the optimal dose regimen has yet to be elucidated.

Many studies considered the planning objective for STAR treatments to be at least 95% of the PTV to be covered by at least 95% of prescription dose. The actual PTV coverage was reported to be in the range of 61–97% [9,19,21,23].

Concerning the planning technique, CyberKnife treatments used fixed cylindrical collimator sizes in the range of 7.5–25 mm or MLC [2,9,16,41]. C-arm linear accelerator treatments most commonly used VMAT plans [4,15,17,20,26,30], but fixed field intensity modulated radiotherapy treatment (IMRT) plans have also been used [4,17]; 6 MV photon beam energy has been used in all but two studies, which used 10 MV FFF [2,29]. More commonly, FFF beam was used due to better OAR sparing and recommendations for patients with ICDs [19,20,34]. Additionally, the reduction in treatment time by increasing the treatment dose rate from 600 MU/min (FF) to 1400 MU/min (FFF) was also considered when using the FFF beams.

#### 2.3.7. Patient-Specific QA

The actual patient targeting accuracy is dependent on pervasive dynamic conditions at patient setup, as well as decreased image quality with the patient anatomy, in contrast to the idealized phantom geometries. Therefore, patient-specific QA should be performed prior treatment delivery in order to verify that the approved treatment plan can be accurately delivered. The patient-specific QA includes certain action levels that would encompass a review of patient setup and patient safety. Specifically, it includes:

- Dose delivery measurements when appropriate;
- Independent check of the approved treatment plan and associated treatment delivery parameters. Verification plans should be created for each treatment plan at the treatment delivery machine, prior to treatment and then perform:

1. Dose measurements with an appropriate phantom;
2. and/or point dose measurements with an ionization chamber in a plastic water phantom, or water-equivalent phantom;
3. and/or planar measurements with electronic portal imaging device-based dosimetry, calibrated for dose response;
4. and/or film dosimetry, with films calibrated for the selected treatment beam.

The physicist team should assess the dose measurements and ensure that they meet the clinical criteria; for the point-dose measurements they should be ±3% of the expected [20]. A gamma analysis should also be performed for the planar measurements. For SBRT, the gamma evaluation criteria of 3%/3 mm, 2%/2 mm or 3%/2 mm are followed (i.e., distance to agreement, DTA = 2 or 3 mm and dose difference, DD = 2 or 3%). Centers follow different reference protocols, therefore, there is no general and standardized threshold for all the centers. At a national survey in Italy, many centers followed the gamma criterion of (2 mm, 2%) >95% to define the plan quality as “ideal”, otherwise as “acceptable” with gamma index (3 mm, 3%) >90% [52]. Knutson et al. followed the criteria of less than 10% of the planar measurements should fail the gamma-index criterion of 2%/2 mm >1, and less than 5% the 3%/3 >1. The reported failing points of 2%/2 mm gamma-index criterion was <8.0% (median 3.0%), <4.3% (median 0.8%) of 3%/3 mm gamma-index criterion in EPID dosimetry, and <4.3% in film measurements.

- Dry run of the approved treatment plan to check for potential collision;
- Verification of patient setup/immobilization.

One of the most potential sources of divergence between planned and delivered dose are positional changes of the target or surrounding tissue. It is possible that the patient position in the immobilization system at treatment is not the same with the position at the time of CT simulation. In order to minimize these potential differences, prior to radiation delivery, the patient is scanned in a body frame, typically a vacuum cushion, with an integral coordinate system that could be visualized in the CT or X-ray image (IGRT system) [34]. The setup error can then be corrected in order to achieve the necessary setup precision required. Stereotactic localization and targeting can be facilitated by a localizer arch, which can be affixed to the body frame or to the couch top, and defines the reference coordinate system. Robotic couches, when used in conjunction with stereotactic X-ray or cone beam computed tomography (CBCT), can also correct for the small rotational errors that can occur (up to 3°–4° for roll and pitch and 10° for yaw) [53].

#### 2.3.8. Treatment Delivery

**Figure 4:**
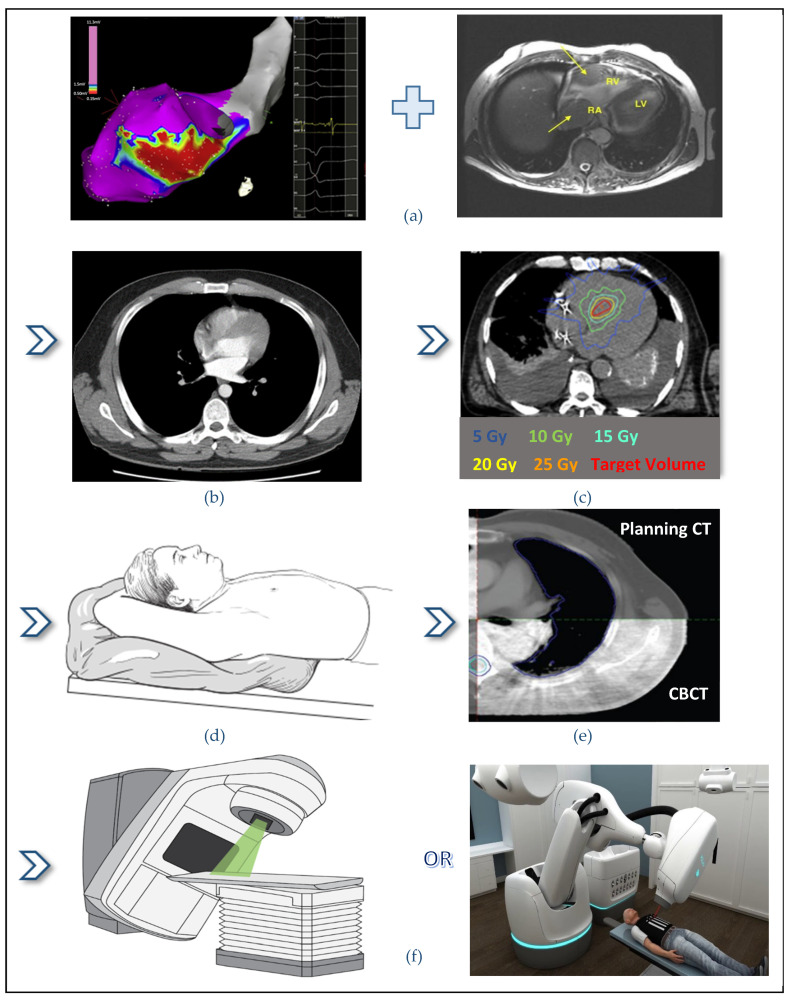
Stereotactic Arrhythmia Radioablation (STAR) workflow: (**a**) Three-dimensional view of the target volume (red) and the surrounding tissues. The electrophysiology information fused with anatomical imaging leads to the target volume generation; (**b**) Planning Computed Tomography (CT) at the CT-simulator; (**c**) Radiotherapy plan showing the target volume and isodose lines. In this example, a dose of 25 Gy in a single fraction is prescribed for delivery to the target volume; (**d**) Patient is positioned and immobilized with the use of a vacuum-assisted device; (**e**) By means of an image-guided, radiotherapy-equipped linear accelerator, a cone-beam CT is used to align the simulation target with the treatment target volume; (**f**) Finally, treatment is delivered with the use of the radiotherapy delivery system. A Linac system on the left and a CyberKnife system on the right are shown.

An example of the STAR treatment workflow, from treatment planning to treatment delivery, is shown graphically in Figure 4. The workflow is similar to thoracic SBRT, but for STAR, additional electrophysiological information is required for the precise delineation of the target volume. Treatment delivery time varies from 4 min to 114 min. C-arm linear accelerator plans are more time-efficient (4–32 min) [4,15,20,27,41] compared to CyberKnife plans (45–114 min) [9,16,19,21,22,25,41]. A pretreatment setup time, for immobilization and alignment of the patient, should also be considered, lasting about 30 min long [2]. Wang et al., who compared three treatment delivery platforms for STAR, reported that CyberKnife plan with InCise 2 collimator had reduced beam delivery time (49% reduction) compared to the beam delivery time using an Iris plan [2].

#### 2.3.9. Follow-Up

Each department performing STAR treatment should apply a follow-up protocol, in order to assess treatment efficacy and safety. Follow-up can include echocardiography, chest radiographs, and clinical follow-up. Quality of life was assessed, usually using the RAND Short Form-36 questionnaire. Table 2 summarizes the follow-up protocols adopted by each group. Case studies showed a remarkable reduction of device-detected VT burden following therapy after a set blanking period. Though, Neuwirth et al. reported a lack of response in two of ten patients and delayed treatment effect in two more patients, which was attributed to smaller target volume and discontinuation of antiarrhythmic drugs before STAR. Follow-up period varied from a limited 6 months, up to 54 months. Neuwirth et al. reported the longest follow-up on safety and efficacy of STAR in patients with structural heart disease and recurrent ventricular tachycardia with a median of 28 months, maximum 54 months [16]. Long-term and thorough follow-up protocols contribute to the optimization of the treatment protocol.

## 3. Discussion and Future Directions

The biggest disadvantage of catheter ablation for ventricular arrhythmias is the limited heating effect to the origin of ventricular arrhythmias in the deep (or mid-) layer of thick ventricular myocardium because these are apart from endocardial or epicardial catheter. On the other hand, STAR is possible to create the appropriate lesions at or near the origin of the arrhythmias regardless of its depth.

Through its advantage, STAR is a relatively novel treatment approach with certain limitations that should be addressed. These include limited number of patients, treatment at a single center, the limited long-term follow-up and narrow “pool” of eligible patients based on current approaches. Additional clinical experience and conduction of a multi-center clinical trial is undoubtedly needed to refine best practices for safety and efficacy. Blanck et al. have published the protocol of a RAVENTA study which aims to be the first study to provide multi-center, multi-platform prospective data on the therapeutic effect of radiosurgery for ventricular tachycardia [49]. Specifically, for AF management with STAR data, it is encouraging, though scarce, because treatment target is small, anatomically not well-defined, and in close proximity to the esophagus, making it challenging to achieve the esophagus dose constraints. Further studies are required to determine feasibility, efficacy, and long-term safety of STAR for treating AF. An ongoing study for AF treatment is NCT04575662 and is expected to publish data for the assessment of STAR safety and AF recurrence for 20 patients [54].

A standardized protocol for the QA process of STAR needs to be defined with an overall goal of extending the duration of treatment effect. This protocol should give guidance for appropriate patient-specific quality assurance processes. Some studies provide information regarding patient-specific quality assurance, but rarely report data beyond general SBRT quality assurance processes [4,15,27]. Knutson et al. reported dosimetric data and their quality assurance process in more detail [20].

Applicability of STAR across all available delivery platforms is also a technical issue. Centers interested in performing this treatment should be equipped with the necessary imaging modalities as well as the appropriate equipment for QA tests in SBRT. Since the heart is moving continuously and thoracic anatomy is very complex, the treatment system must be very precise and able to track moving targets. Availability of ECGI is also important in order to allow direct registration of pictures from electroanatomical mapping and computed tomography. Direct registration of the above-mentioned imaging methods with the ability to contour precisely the target volume, which is an actively moving target, is extremely helpful. Close collaboration among radiation oncologists, electrophysiologists, cardiac imaging specialists, and medical physicists is paramount for the treatment to achieve high levels of ablation accuracy and help in progressing this technique.

Studies with long-term follow-up are expected in order to provide data regarding safety and efficacy, and to document long-term toxic effect of radiotherapy on cardiac structures, as is well documented for treatment of lymphomas [55]. The underlying mechanism of radiation-associated treatment effect and its long-term durability needs to be elucidated, as the mechanism of injury for high doses of radiotherapy is less well known [56]. A possible mechanism of ablative radiotherapy is a combination of vascular injury, leading to tissue hypoxia or necrosis, and apoptotic cell death, resulting in fibrosis and scar formation. However, these postulates relate to radioablative injury in malignant tumor cells. Even less is known about the cellular injury mechanisms of the nonmalignant arrhythmogenic cardiac tissues [57]. Another issue arising from the available clinical data is that patients selected for STAR had end-stage disease, with a low likelihood of survival free from further VT episodes for which conventional therapy has already failed. Gianni et al., in a prospective study of five patients, reported that the negative outcome of STAR was the short-term efficacy [19]. Nevertheless, they clarify that the cohort of patients had advanced heart failure. Scar progression is expected in that population, which can explain the formation of new arrhythmogenic substrate and the long-term new ventricular arrhythmias. Consequently, it is not known how effective cardiac SBRT is for patients with less refractory disease.

Long-term data will also define the full safety profile of STAR for toxicities on healthy intra-cardiac structures, such as the coronary arteries. Most studies used OAR dose constraints based on hypo-fractionated centrally located lung cancer treatments, which generally comprised of extra-cardiac OAR constraints [5,17,34,44]. Dose limits to heart substructures are largely unknown and have only been recently reported, though not yet correlated to toxicity [20,49]. At the RAVENTA trial, cardiac substructure contours were delineated and dose limits were classified to minor and major violations [49]. Specifically, the major violation for left coronary arteries was set to 20 Gy. Patients receiving coronary arteries with a dose >20 Gy were excluded from the trial (PTV was close or overlapping with coronary arteries). So far, there has been no evidence of short-term toxicities in the coronary arteries, but patients are being followed up after study completion in order to obtain further long-term safety data.

Dose prescription is another clinical question under consideration. The dose of 25 Gy was adopted from the first human case report by Zei et al. [58]. This dose was considered safe and followed by almost all studies, except Zeng et al., who delivered 24 Gy in three fractions [23]. Chin et al. performed STAR with lower doses of 15 and 20 Gy, when the normal tissue constraints could not be met with the standard dose of 25 Gy, and reported also one patient who received safely a second round of SBRT [18]. Higher dosing was found to be safe in preclinical studies. Dose escalation above 25 Gy leads to scar formation so the treatment might be more effective, as reported by Blanck et al. [59]. Dosages of up to 35 to 40 Gy had no complications associated with radiation [14,51]. Further research studies could elucidate the dose issue and move this extremely promising area of functional radiosurgery a step forward.

Finally, a study from Dusi et al. reported that proton radiation therapy may be a step forward [60]. Particle therapy has a theoretical advantage over photon beams due to the Bragg peak; a higher dose at the end of the particle range, potentially allowing for a high dose deposition on very small cardiac targets. The comparison of simulation plans with protons instead of photons, in a phase I/II prospective study in 17 patients [4], showed a significant sparing in non-target heart tissue (mean dose 1.9 Gy as compared to 6.2 Gy), while meeting the organs-at-risk constraints. They refer, though, that the problem of breathing and cardiac movements may be even greater in particles, and target irradiation with small spots could produce hot or cold spots. Concluding, particle therapy may potentially offer advantages over photons, but several challenges are present.

## 4. Conclusions

In conclusion, STAR seems to be a promising treatment for refractory scar-related VT and drug-refractory AF, though AF treatment presents different challenges due to the anatomical arrhythmogenic tissue site. STAR has been reported to result in a remarkable reduction in the burden of VT and no recurrence of AF in most patients, as well as improved quality of life. Nevertheless, it is currently a bail-out procedure for patients not responding to conventional treatment. Despite its limitations, non-invasive SABR has undoubtedly extended the therapeutic horizon for ventricular arrhythmias and clearly warrants further investigation to establish its mechanism of action, universally accepted protocols, and long-term efficacy and safety.

## Figures and Tables

**Table 1:** Patient characteristics and inclusion criteria for STAR treatment.

Study	No. of Patients	Patient Characteristics	Cardiomyopathy Type	Inclusion Criteria				Exclusion Criteria
				Episodes of VT	Antiarrhythmics *	History of Catheter Ablation	Additional	
Cuculich, P. 2017 [15]	5	High-risk, refractory VT ICD present Mean LVEF = 23%	NICM (3 pts) ICM (2 pts)	≥3	≥2	≥1 or contraindication	-	LVAD
Robinson, C. 2019 [4]	19	Treat-ment-refractory episodes of VT PVCs-related cardiomyo-pathy Median LVEF 25%	17 for VT PVC-induced cardiomyopathy (2 pts) ICM (11 pts) NICM (8 pts)	≥3	≥1	≥1 or contraindication	≥18 years old	Past radiotherapy to the anticipated treatment field Inotrope- dependent heart failure LVAD Expected survival <12 months Polymorphic VT or ventricular fibrillation >3 distinct clinical VT morphologies, or >5 induced VT morphologies
Cvek, J. 2014 [21]	1	Recur-rent VT Recur-rent arrhythmic storm LVEF = 25%	NICM (case report)	–	–	Ineffective due to excessive myocardium thickness	All standard treatment options exhausted	N/A
Loo, B.W. 2015 [22]	11	Coron-ary artery disease Coron-ary artery bypass LVEF = 24% ICD present	N/A	–	–	Contraindicated	–	N/A
Neuwirth, R. 2019 [16]	10	Recurrent scar-related VTs Mean LVEF = 26.5	NICM (2 pts) ICM (8 pts)	–	–	Failed due to Inaccess-ibility of the critical substrate part	–	Subjects with mechanical assist devices
Zeng, L.J. 2019 [23]	1	Refractory VT secondary to unresectable cardiac lipoma	N/A	–	Failed	Failed	–	N/A
Haskova, J. 201 [24]	1	Repetitive monomorphic VTs of several morphologies Unresectable cardiac fibroma ICD present	N/A	Occurr-ence of incessant VT	–	Failed	–	N/A
Jumeau, R. 2018 [25]	3 (report-ed: 1)	Dilated cardiomyo-pathy of unspecified origin ICD present LVEF = 15%	NICM (1 reported pt)	–	Unrespon-sive	Unrespon-sive	–	N/A
Bhaskaran, A. 2019 [26]	1	VT storm	N/A	–	Unresp- onsive	Unrespon- sive	–	N/A
Lloyd, M.S. 2019 [17]	10	Refractory VT	NICM (6 pts) ICM (4 pts)	–	≥2	≥1	Failed 1 adjunctive therapy (mechanical support or sympathetic blockade)	N/A
Chin, R. 2020 [18]	8	Refractory, scar-related VT	NICM (4 pts) ICM (4 pts)	–	–	Failed multiple RF ablations or contra-indicated	–	N/A
Gianni C. 2020 [19]	5	Refractory, scar-related VT LVEF = 34%	NICM (1 pt) ICM (4 pts)	Recurrent sympto-matic VT that induced ICD shock(s)	–	–	ICD present Age ≥60 LVEF ≥20%	Prior radiation therapy to the chest Active ischemia or other reversible VT causes Expected survival <12 months Baseline creatinine levels >1.5 mg/dL
Krug D. 2019 [27]	1	Dilated cardiomyopathy LVEF = 15%	N/A	–	–	Contraindicated	–	N/A
Scholz, E.P. 2019 [30]	1	Ventricular fibrillation storm	–	–	–	–	All available cardiologic therapeutic strategies failed	–
Monroy, E. 2016 [31]	1	Paroxysmal AF	–	–	Adverse effects of the AAD and failure to alleviate the symptoms	Contraind-ication	–	–
Qian, P.C. 2020 [9]	2	Drug-refractory paroxysmal AF	–	–	≥1	Refused catheter ablation of AF	Symptom-atic AF	Inability to provide informed consent Age <18 years Contraindication for RT including prior RT to the same body region Pregnancy or significant vasculitides or autoimmune disease Contraindication for anticoagulation, NYHA class IV congestive heart failure Medical prognosis <6 months
Shoji, M. 2020 [32]	3	Drug-refractory AF	–	–	≥1	Unsuitable candidates	Diagnosis of metastatic cancer No history of surgical treatment of AF Age 20–85	Acute myocardial infarction within 8 weeks before enrollment Pregnancy Creatinine value >1.3 mg/dL Second or third degree atrioventricular block LVEF <35%

AAD = Antiarrhythmic Drugs, AF = Atrial Fibrillation, ICD = Implantable Cardioverter-Defibrillator, ICM = Ischemic Cardiomyopathy, LVAD = Left Ventricular Assist Device, LVEF = Left Ventricular Ejection Fraction, N/A = Not Available, NICM = Non-Ischemic Cardiomyopathy, pts = Patients, PVC: Premature Ventricular Contraction, RF: Radio Frequency, RT: Radiation Therapy, VT: Ventricular Tachycardia. * Failed, intolerance or declined.

**Table 2:** Safety end points and the corresponding monitoring methods for the patients of each study.

Study	Safety Endpoints	Monitoring (Follow-Up)	Outcomes
Cuculich, P. 2017 [15]	Complications	Adjudication of all ICD interrogations TTE at baseline and at 1, 6, and 12 months after treatment Chest CT at baseline and at 3 and 12 months	No complications during the treatment Mean LVEF did not decrease with treatment At 3 months, adjacent lung showed opacities consistent with mild inflammatory changes, which had resolved by 1 year
Robinson, C. 2019 [4]	The rate of ≤90-day SAEs defined using CTCAEs	ICDs remotely monitored and at each study visit Study visits at: day 3; at 2, 4, and 6 weeks; at 6 and 12 months, and annually thereafter Oral anticoagulation prescribed during the first month after treatment 12-lead ECG At: day 3; at 6 weeks, and at 3, 6, and 12 months Chest CT along with ECGI at: 3 and 12 months For PVC patients a 24 h Holter monitor performed at: week 6 and at months 3, 6, and 12	In the first 90 days, 2/19 patients (10.5%) developed a treatment-related SAE No acute toxicity
Cvek, J. 2014 [21]	Signs of toxicity	ECG monitoring ICD monitoring	10 days after treatment: minimal elevation of troponin T serum level After six weeks: no complications or side effects
Loo, B.W. 2015 [22]	Complications	ICD monitoring 2.5 months post-STAR and PET-CT TTE at 1, 3, and 6 months	No definite acute or late complications
Neuwirth, R. 2019 [16]	Acute and late radiation-induced events according to the CTCAE 4.0 scale	TTE and ICD interrogation at: 1, 3, 6, and 12 weeks after STAR, and at 3-month intervals thereafter Plasma troponin levels at the first four clinical visits NT-pro-BNP: at 1 week, 3 months, and 1 year CXR	Acute radiation-related toxicity at 4 pts: nausea One possible late cardiac radiation-related toxicity, grade 3, 17 months after STAR
Zeng, L.J. 2019 [23]	Complications	Not mentioned	No complications during 4 months of follow-up
Jumeau, R. 2018 [25]	Complications	Not mentioned	No complications
Bhaskaran, A. 2019 [26]	Complications	Holter monitoring TTE	No complications during 2 months of follow-up
Lloyd, MS. 2019 [17]	Acute and late radiation-induced events	ICD monitoring 3 months post-STAR for: VT detection total ICD shocks, and total (ATP) sequences	1 patient experienced a slow VT below the treatment zone of her device during SBRT and was resuscitated 2 patients did exhibit signs of low-grade pneumonitis within 3 months of follow-up
Chin, R. 2020 [18]	Complications	ICD interrogation at: 1, 3, 6, and 12 months or to the time of death after SBRT Screening at each visit for the development of radiation therapy-related organ toxicities TTE obtained 3 months after SBRT	No acute complications No definite organ toxicities during follow-up No adverse outcomes related to ICD function
Gianni, C. 2020 [19]	Radiation-related adverse events	ICD interrogation at: 2 weeks; 1, 2, 3, 6, 9, and 12 months after STAR TTE CXR Chest CT scan	No clinical or imaging evidence of radiation-induced complications in the organs at risk
Krug, D. 201 [27]	Radiation-related adverse events	ICD interrogation Histopathologic examination of the radiosurgery target region TTE	Elevated cardiac enzymes Nausea at the day of treatment No other radiation-induced side effects
Scholz, E.P. 2019 [30]	Acute and late radiation-induced events	Holter monitoring TTE	No complications during 2 months follow-up
Monroy, E. 2016 [31]	Acute and late radiation-induced events	Cardiac MRI using late gadolinium enhancement Clinical evaluation every 4 to 8 weeks until 6 months after therapy	No esophageal, respiratory, or pericardial symptoms
Qian, P.C. 2020 [9]	Acute and late radiation-induced events	Monthly history, examination ECG TTE Chest CT Cardiac MRI within the first 12 months	No immediate physical complaints nor observed complications No adverse clinical effects 3 years after therapy
Shoji, M. 2020 [32]	Complications	ECG Transesophageal echocardiogram TTE	No immediate physical complaints nor observed complications No evidence of radiation-related complications

ATP: Antitachycardia Pacing, CT: Computed Tomography, CTCAEs: Common Terminology Criteria for Adverse Events, CXR: Chest X-ray, ECG: Electrocardiography, ECGI: Electrocardiographic Imaging, ICD = Implantable Cardioverter-Defibrillator, LVEF: Left Ventricular Ejection Fraction, MRI: Magnetic Resonance Imaging, NT-pro-BNP: N-Terminal prohormone of Brain Natriuretic Peptide, PVC: Premature Ventricular Contraction, SAEs: Serious Adverse Events, SBRT: Stereotactic Body Radiation Therapy, STAR: Stereotactic Arrhythmia Radioablation, TTE: Transthoracic Echocardiography, VT: Ventricular Tachycardia.

## Data Availability

Not applicable.
